# Impact of the Variety of Tef [*Eragrostis tef* (Zucc.) Trotter] on Physical, Sensorial and Nutritional Properties of Gluten-Free Breads

**DOI:** 10.3390/foods11071017

**Published:** 2022-03-30

**Authors:** Marina Villanueva, Workineh Abebe, Sandra Pérez-Quirce, Felicidad Ronda

**Affiliations:** 1 Department of Agriculture and Forestry Engineering, Food Technology, College of Agricultural and Forestry Engineering, University of Valladolid, Av. Madrid, 44, 34004 Palencia, Spain; marina.villanueva@uva.es (M.V.); workinehabebe.zeleke@uva.es (W.A.); pqsandra@hotmail.com (S.P.-Q.); 2 Ethiopian Institute of Agricultural Research, Addis Ababa P.O. Box 2003, Ethiopia

**Keywords:** tef varieties, in vitro starch digestibility, mineral content, sensory analysis

## Abstract

Tef is currently being incorporated into a wide range of foodstuff due to its high nutritional profile. This study tries to fill the information gap on the effect of tef varieties on physico-chemical, nutritional and sensorial quality of gluten-free bread. Maize starch replacement at 50, 75 and 100 g/100 g level by tef flour from three Ethiopian varieties (DZ-Cr-37, DZ-Cr-387 and DZ-01-99) resulted in viable gluten-free breads with acceptable sensory properties, higher mineral content and lower glycemic response. Tef cultivar type and blending level significantly affected bread quality. The 50% and 75% substitution levels and the DZ-Cr-37 variety led to the highest bread volumes with the lowest firmness. Breads made with DZ-01-99 variety were darker and with more reddish crust and crumb hues than those made with the other varieties. Breads from 100% DZ-Cr-37 achieved the highest hedonic scores for color, odor, taste and texture. The Ca, Fe and Mg contents of the breads made with 100% tef were 13, 40 and 30 times, respectively, higher than those of the control bread (100% maize starch), indicating tef could be used as an excellent source of these important minerals. In addition, the rapidly digestible starch content decreased up to 28% in breads fortified with tef flour.

## 1. Introduction

Several studies indicated that the current gluten-free (GF) diet is mainly based on products from rice and maize [1]. However, sticking to such a diet can cause lack of appropriate nutrition such as deficiencies of several vitamins and minerals and malabsorption of nutrients in celiac subjects [2,3]. Therefore, more attention is being given to high-nutritional-profile ancient GF grains, such as tef, a suitable substitute for wheat especially for people with celiac disease, gluten allergy or gluten sensitivity as well as people who choose to follow GF diet [2]. Products from tef are rich in dietary fiber that is high in an insoluble fraction, as it is utilized as whole flour. It presents good amino acid composition and high mineral content [4,5]. Its high iron content is one of the most important attributes that could help to reduce the iron deficiency that is usually observed in celiac patients [6]. In addition, tef has slow-release carbohydrates, which makes it recommendable for type 2 diabetes patients [7]. It is also rich in varieties of bioactive compounds, such as phytosterols, vitamins and phenolic compounds, which contribute to the promotion of human health [8].

Application of tef in the formulation of more nutritious and healthier GF breads has been studied by different researchers in the past. Sourdough technology was applied in order to improve the sensorial quality of gluten-free breads from tef [9]. The potential for improving the quality of tef-based GF bread by enhancing their protein composite networks through the incorporation of enzymes and hydrocolloids was also evaluated [10,11]. These studies were limited to commercially available tef types (white and brown), and there is a lack of information on the impact of tef variety type and incorporation level in GF bread formulations.

Previous studies demonstrated that the physico-chemical and nutritional quality of tef flours and products formulated from them are importantly affected by the type of tef variety [12,13,14,15]. Analysis done on gel viscoelastic properties of three tef varieties, DZ-Cr-37, DZ-Cr-387 and DZ-01-99, at five incorporation rates, 6, 8, 10, 12 and 14% *w/w*, revealed that gels made from DZ-01-99 had higher elastic and viscous moduli and tolerated higher shear stress without breaking [16] than the gels made from the other two varieties. Ronda et al. [17], employing the same varieties of tef in chapatti-type wheat-based bread formulation, found that the incorporation of up to 30% of flour from the two white tef varieties (DZ-Cr-37, DZ-Cr-387) did not have a detrimental impact on either loaf volume or crumb hardness and cohesiveness of the bread, while the use of the brown DZ-01-99 variety increased the bread volume by 10% compared to the control bread (100% wheat). They also found higher flavonoids content and anti-radical activity in the bread enriched with DZ-01-99 (brown tef) flour. On the other side, wheat bread doughs supplemented with the DZ-Cr-37 variety exhibited the highest elastic and viscous moduli, the lowest compliances and the highest steady state viscosity and led to significantly lower volume of bread [13]. Our recent study demonstrated the procedure to make technologically feasible and sensorially acceptable gluten-free bread from tef flour [15]. This study also revealed that the type of tef variety and its level of addition significantly affected the viscoelastic properties of GF doughs that influenced bread volume and grain structure of the crumb [15]. However, there is still a lack of information about the influence of the tef variety and level of addition on the physical and sensory properties as well as on the nutritional value, with regard to the mineral content and in vitro glycemic index of GF breads, that the present work tries to address.

## 2. Materials and Methods

### 2.1. Raw Materials and Chemicals

Three types of tef varieties, DZ-01-99 (brown tef), DZ-Cr-37 (white tef) and DZ-Cr-387 (white tef), were collected from the Debre Zeit Agricultural Research Center of the Ethiopian Institute of Agricultural Research (EIAR). The tef grains were whole floured at a cottage grain mill house (Bishoftu, Ethiopia) that utilized a two-disk attrition mill. Then, the flours were quickly packed in airtight plastic bags and stored at 4 °C until analysis. AACC method 44-10 was used to determine the moisture contents (mc) of the tef flour samples, and the results recorded were 12.02 g/100 g, 11.80 g/100 g and 10.99 g/100 g for DZ-Cr-37, DZ-Cr-387 and DZ-01-99, respectively. Maize starch was supplied by Ferrer Alimentación S.A. (Barcelona, Spain) (11.21 g/100 g of mc). Sunflower oil, sugar and salt were purchased from the local market and hydroxypropylmethylcellulose (HPMC, Methocel K4M Food Grade) was provided by Dow Chemical (Midland, TX, USA). All the chemicals utilized were reagent grade.

### 2.2. Bread Making

A straight dough process was employed to make the breads, and the formula was on a 100 g maize starch or maize starch + tef flour (14 g/100 g of mc) basis: 5 g sugar, 1.5 g salt, 2 g HPMC, 6 g of oil, 3 g dried yeast and 90 g water. Tef flour was incorporated at levels of 50 g, 75 g and 100 g/100 g of mixture. Maize starch and tef flour were blended with a Chopin MR2L/MR19L mixer (Chopin technologies, France) for 20 min. The dough was prepared in a KitchenAid professional mixer (KPM5) by mixing the solid ingredients first for 2 min at speed 1, and then oil was added and mixed with solid ingredients for 2 min at speed 2. A two-phase kneading process was undertaken: at speed 2 for 4 min adding water with dissolved yeast during the first minute and at speed 4 for an additional 4 min phase. Proofing was done by putting 200 g of dough into single-use aluminum pans for 40 min at 28 °C and 85% relative humidity, and baking was carried out in a Sveba Dahlen (Fristad, Sweden) at 170 °C for 20 min. Then, before bread quality analysis, the baked breads were allowed to cool at room temperature for 1 h. Two batches of each formulation were prepared for analysis, and each batch included six loaves of bread.

### 2.3. Bread Quality Evaluation

The volume of the breads was determined in duplicate utilizing a Volscan profiler 300 (Stable Microsystems, Surrey, UK). The weight of the bread samples was immediately recorded after cooling to determine the baking loss, by subtracting the weight of the bread from the initial weight of the dough, and the specific volume, by dividing the bread volume by the bread weight. Minolta spectrophotometer CN-508i (Minolta, Co. LTD, Tokyo Japan) was employed to measure bread crumb and crust colors, and results were expressed in the CIE L*a*b* and CIE L*C*h coordinates using the D65 standard illuminant and the 2° standard observer. The measurement of color was done 5 × 5 times (5 different points on each bread and 5 times on every point). A TA-XT2 texture analyzer (Stable Microsystems, Surrey, UK) utilizing “Texture Expert” software was used to measure crumb texture. Texture Profile Analysis (TPA) with double compression test penetrating 50% of the depth of the crumbs of 20 mm thick bread slice samples at 1 mm/s speed test, with a 30 s delay between the first and second compression, was applied using a 20 mm diameter aluminum cylindrical probe. Firmness (N), springiness, cohesiveness, chewiness (N) and resilience were calculated from the TPA chart. Analysis was carried out on two loaves from each batch and on two slices taken from the center of each loaf.

### 2.4. Sensory Evaluation

Multi-sample difference test recommended in ISO 6658:2017, with some modification suggested by Meilgaard et al. [18], was applied to undertake the hedonic sensory evaluation of bread. The study included 127 untrained, non-celiac bread consumer volunteers, 54% males and 46% females, with ages ranging between 15–64 with a mean ± standard deviation of 24 ± 13 years and with a random socioeconomic background. Panelists rated the crust color, crumb color, odor, taste, aftertaste and texture of the breads on a non-structured scale ranging from 0 (I like it much less than R) to 10 (I like it much more than R), where R was a reference bread that was positioned in the middle of the scale. An arbitrary score of 5 was given to the reference bread (R), and it was a GF bread made with 60 g rice flour and 40 g maize starch per 100 g of the mixture, following the same procedure described for the remaining bread samples (see Section 2.2). Panelists were offered three-digit-coded pieces of 40 mm × 40 mm of the bread loaves made with 50 g, 75 g and 100 g/100 g tef flour of the three varieties in three sessions. Panelists were offered water to rinse the mouth between samples. The control bread (100 g/100 g maize starch) was included in each session. The R bread (60 g rice flour and 40 g maize starch per 100 g of mixture) was included and used by panelists as a reference to locate the center of the scale because it represents a common formulation of commercial GF breads with an acceptable quality. The 100% maize starch bread was not used as a reference due to its poor quality. Before starting the analysis, all the volunteers were informed of the aim of the sensory evaluation, and they gave us the informed consent to perform the evaluation. The guidelines of the European Group on Ethics in Science and New Technologies were followed during the sensory analysis.

### 2.5. Mineral Content

The minerals (Ca, Cr, Cu, Fe, K, Mg, Mn, P and Zn) available in the flours and bread samples were quantified by employing a Radial Simultaneous inductively coupled plasma optical emission spectrometry (ICP-OES) Varian 725-ES spectrophotometer (Agilent Technologies, Santa Clara, CA, US). Aliquots of samples (0.5 g) were put in Teflon cups, diluted with 6 mL of 65% HNO_3_ and 2 mL of 30% H_2_O_2_, heated up to 200 °C for 6 min and held at 200 °C for 15 min in a microwave digester (MLS 1200 mega, Milestone, Shelton, CN, US) for the mineralization and finally diluted to 25 mL. The mineral content determinations in each sample were carried out in duplicate.

### 2.6. Starch Fractions Analysis

In vitro starch digestibility and the free sugar glucose (FSG) contents of the formulated breads were determined as described in Englyst et al. [19] with some changes suggested by Solaesa et al. [20]. The amount of glucose freed at 20 min (G20) and 120 min (G120) and the total glucose (TG) were measured by the glucose oxidase colorimetric method. Then, rapidly digestible starch (RDS) = 0.9·(G20 − FSG), slowly digestible starch (SDS) = 0.9·(G120 − G20), total starch (TS) = 0.9·(TG − FSG), rapidly available glucose (RAG) = G20 and total digestible starch (TDS) = RDS + SDS were calculated. Each sample was measured at least in triplicate.

### 2.7. Statistical Analysis

Data analysis was done by using multifactor analysis of variance analysis (ANOVA), and means were compared (*p* < 0.05) with Fisher’s least significant difference (LSD) test using Statgraphics XVIII software (Bitstream, Cambridge, MN, USA). Pearson correlation matrix was also applied.

## 3. Results

### 3.1. Bread Quality

**Figure 1:**
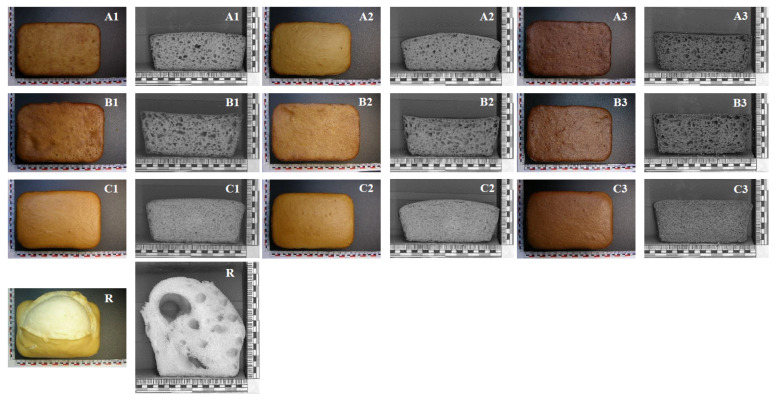
The influence of tef flour incorporation level and variety on the appearance of GF breads. Addition levels: (**A**): 100 g tef flour per 100 g of mixture; (**B**): 75 g tef flour per 100 g of mixture; (**C**): 50 g tef flour per 100 g of mixture; tef varieties: 1: DZ-Cr-37; 2: DZ-Cr-387; 3: DZ-01-99; (**R**): 100 g/100 g maize starch bread.

Bread made with 100% maize starch showed important crumb structure deficiencies, as can be seen in Figure 1, which made it difficult to measure its physical quality parameters. Horstmann et al. [21] also reported big holes in the structure of maize starch breads. They hypothesized that lower lipid content caused less stability of network interfaces, leading to partial network breakdown resulting in these deficiencies. The substitution of maize starch by tef flour improved the appearance of breads (Figure 1), with an influence of the doses in the trends of bread volume and crumb structure for the three tef varieties. Accordingly, the specific volume and textural parameters of the breads were also dependent on these factors (Table 1).

The bake loss ranged between 13% (100% DZ-Cr-37) and 17% (75% DZ-Cr-387). The 75% tef-incorporated breads had the highest values. Bake loss is positively related to the loaf volume of the loaves that gives higher exchange surface area for weight loss. However, in this study, no correlation was found between volume and bake loss. The probable reason for this could be attributed to the different flour hydration properties of the tef varieties reported by Abebe et al. [12].

The dilution of tef flour with maize starch resulted in a marked improvement in the specific volume of bread (Table 1), which was significantly affected (*p* < 0.001) by tef incorporation level, tef variety type and their interaction (variety type x level). Bread baked from 100% tef flour, regardless of the variety, showed a lower specific volume than those formulated from the mixture of tef flour and maize starch. The lowest value (1.71 mL/g) was obtained for the bread made from 100% DZ-Cr-387 variety. The high consistency of bread doughs made only with tef flour would explain this result [15]. It would hinder the development of the dough under the action of the tension exerted by the gas formed during fermentation and its expansion by the time of baking, leading to loaves of smaller volume [22]. The higher water absorption capacity of tef flour (with protein and fiber) compared to maize starch [23] could be the reason for the lower amount of free water in the dough and its higher consistency. The higher specific volumes were obtained at 75% and 50% doses of DZ-Cr-37 and 50% of DZ-01-99, where the values ranged between 2.42 and 2.50 mL/g. The incorporation of maize starch into tef flour resulted in an optimal consistency of the dough, allowing a higher expansion of the dough while at the same time promoting the retention of the gas produced during fermentation and preventing its coalescence and loss during both fermentation and baking (as happened in 100% maize starch bread), allowing a higher volume of bread [15]. Previous works showed that the addition of up to 30% tef grain flours did not have a detrimental effect on wheat-based loaf volume. This controlled addition even provided an increase of 10% with respect to the control bread (100% wheat) when brown grain tef flour (DZ-01-99) was used. However, further incorporation of tef flour (from 30 to 40%), regardless the tef variety, had deleterious effects in terms of decreasing loaf volume [17].

The blend of tef flour with maize starch resulted in a marked improvement in the textural qualities concomitant to the specific volume of the breads. Except for springiness, the effects of tef flour incorporation level, variety type and their interaction were significant (*p* < 0.05) on all the measured textural parameters (Table 1). As expected, a negative significant correlation (*p* < 0.01) was obtained between the specific volume and crumb firmness (*r* = −0.84), cohesiveness (*r* = −0.78), chewiness (*r* = −0.85) and resilience (*r* = −0.81). The increase in bread firmness with the increase of tef flour dose from 50% to 100% was more pronounced in DZ-Cr-99 (+103%) incorporated breads than DZ-Cr-37 (+80%) and DZ-Cr-387 (+66%). This corroborated the report by Ronda et al. [17] in ciabatta type wheat-tef breads where the hardness scored by 40% DZ-Cr-99 incorporated breads stood relatively higher than those of 40% DZ-Cr-37 and 40% DZ-Cr-387 ones.

**Figure 2:**
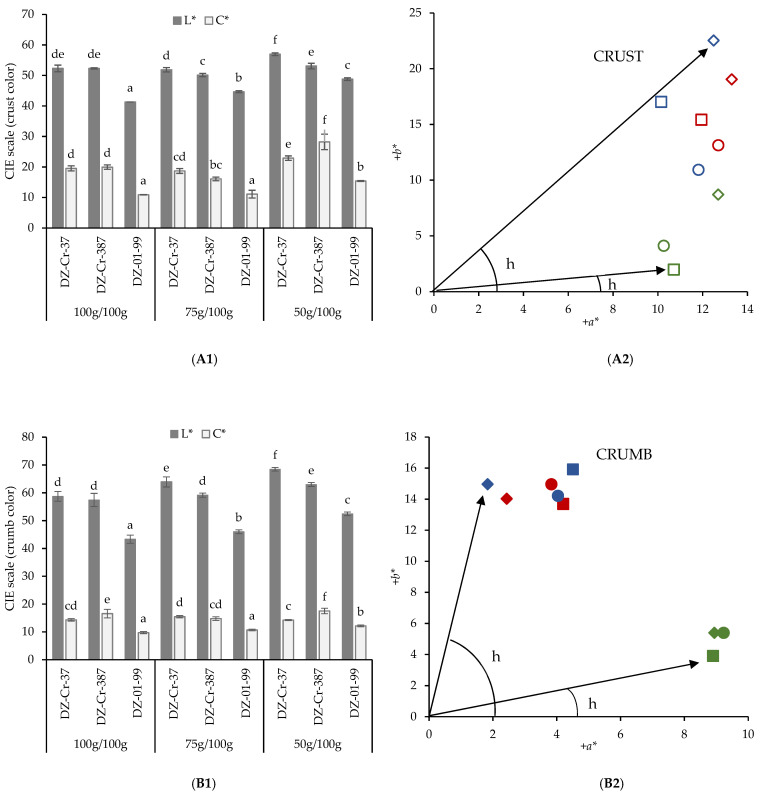
Influence of tef flour addition level and variety on crust (**A**) and crumb (**B**) bread color. Crust and crumb lightness and chroma (**A1**,**B1**) and hue (**A2**,**B2**) of maize starch/tef flour GF breads. Red symbols represent breads made with the DZ-Cr-37 variety, blue symbols the DZ-Cr-387 variety and green symbols, the breads made with DZ-01-99 variety. Squares represent the 100 g/100 g dose, circles represent the 75 g/100 g dose and diamonds, the 50 g/100 g dose. The hue of the indicated samples (located at extreme angular positions in the chromatic diagram) is represented by the angle identified as h. In Figures (**A1**) or (**B1**) columns of the same series with a letter in common correspond to values that are not significantly different (*p* > 0.05).

White grain tef varieties showed higher values of crumb and crust lightness (L*), chroma (C*) and hue (h) than breads made from the brown tef (DZ-01-99) (Figure 1 and Figure 2). Lightness (L*) significantly decreased with an increasing level of tef in the formulation. L* values decreased from 53 to 43 (crumb) and 49 to 41 (crust) in DZ-01-99 breads as the level of incorporation increased from 50% to 100%, denoting darker bread crumb and crust. In the white grain tef breads, a slight decline in crumb lightness was also observed with the highest addition of tef flour, ranging from −5.6% (DZ-Cr-387) to −8.9% (DZ-Cr-37). Similar decreases were also found on slice lightness with grain tef flour addition in earlier works, and this could be attributed to bran particles in wholegrain flours that led to a darker crumb color [11,17]. The hue of the crumb, in which the color is mainly associated to the original color of ingredients, decreased gradually with the addition of tef flour, up to 55% for DZ-01-99 brown tef variety, 12% with DZ-Cr-387 variety and 9% with DZ-Cr-37 variety. This decrease means a more reddish crumb of the bread more enriched in tef flour.

### 3.2. Sensory Evaluation

The effects of tef flour addition on bread sensory analysis are presented in Table 2. In general, tef incorporation provided scores similar to the reference. The panelists showed a slight dissatisfaction in the color of the crust and crumb of the breads evaluated with respect to the reference. Even so, there were breads with scores above 5 on the ten-point scale. Breads made with 100% DZ-Cr-37 variety and with 75% DZ-Cr-37 and 75% DZ-Cr-387 varieties obtained the best scores for both crust (5.5, 5.1 and 5.3, respectively) and crumb color (5.6, 5.3 and 5.2, respectively). In general, these three breads together with 50% of the variety DZ-01-99 were the ones with the best scores also in terms of odor, taste and aftertaste. On these attributes, white tef varieties were more appreciated than brown ones, admitting higher doses of addition (75% vs. 50%). The higher polyphenols and the particular volatile profiles content in brown tef that produce higher bitterness [17,24] may be related to this fact.

Other authors reported the negative evaluation obtained by tef breads due to the absence of intensities of yeast, whereas the dough-like, malty and buttery attributes were found as positives in wheat crumb [10]. However, a study on the partial substitution of corn starch and rice flour bread mix by tef, amaranth and quinoa flour showed that breads containing tef were better valued in almost all attributes compared to corn starch/rice flour mixture [25].

The 100% tef breads, regardless the variety, were better rated in terms of sensorial texture (5.1 for the DZ-01-99 and 5.5 for the two white varieties) than the breads made with lower tef incorporation level, in spite of the fact that they showed the lowest specific volume and the highest instrumentally measured firmness. The most likely reason is the mouthfeel of their denser and more cohesive crumbs, which resulted in a more pleasant texture compared to the other two doses studied, with lighter and crumblier crumbs. Ronda et al. [17] reported that wheat breads with up to 40% of tef flour from the same varieties used in this work were judged acceptable (scores higher than 5) for appearance, odor, texture, taste, persistency and overall acceptability. Other authors obtained higher values of crust color (7.4), aroma (7.5) and general acceptance (7.1) for gluten-free breads made with a commercial GF bread mix and 25% of tef bread [25]. Our results confirm that breads can be made with 100% tef flour with good sensory quality, if the appropriate tef variety is chosen.

### 3.3. Mineral Content

Table 3 shows the macro and microelement content obtained for bread made with the three different tef varieties and the maize-starch control bread. The table also includes the minerals content of the tef flours and the maize starch utilized in the study. Considering the moisture content of 100% tef breads (~50%), it can be concluded that the mineral content of breads, expressed as dry matter, was very similar to that obtained for tef flour. In agreement with previous works [5,17], the mineral content of tef flour was notably higher than that of maize starch or other cereal flours [26]. Although Ronda et al. [17] studied the same varieties of tef, the results differed. This could be because the mineral content of tef flours is very dependent on environmental factors [27], and the harvest year of the grains used in both works was different.

In general, it can be observed that the bread mineral content showed a decreasing trend with reducing tef flour dosage. Breads with 50% tef flour showed half the mineral content of those made with 100% tef flour. The ANOVA indicated a significant effect of the tef incorporation level (*p* < 0.001) on all the elements studied while the tef variety did not show any effect. The differences among the three tef varieties studied were more pronounced at the 100% level than 50%. At the highest incorporation level, the DZ-01-99 variety stood out for its high Ca, K, Mg, Mn and P contents, while the DZ-Cr-387 variety showed higher values of Cu and Fe. In terms of the minerals analyzed in this study, the most popular GF raw materials (rice, maize and GF wheat starch) are inferior to the less popular cereals like tef [25]. The Ca, Fe and Mg content of 100% tef breads were 15, 44 and 33 times, respectively, higher than that of the control bread (100% maize starch), revealing that tef is an excellent source of these important macro-minerals. The K and P contents in the control bread were 34 mg/100 g and 28 mg/100 g, respectively, while 100% tef breads were 268 mg/100 g and 245 g/100 g (on average, regardless of the tef variety type), respectively, which represents an increase of around 750%. This result corroborates the recommendation by Ronda et al. [17] that, on the basis of Recommended Dietary Allowances (RDA), adequate intake of such minerals can easily be met through daily consumption of gluten-free breads formulated from tef. Based on the current study, daily consumption of 100 g of bread made with 100% tef could satisfy 50–75% of female requirements and fully cover male adequate intake.

### 3.4. Starch Fractions of Breads

Recently, different strategies have been proposed to reduce the risks of diseases associated with the increased level of sugars in the blood. Table 4 shows the starch fractions obtained according to their capacity to be hydrolyzed to glucose by digestive enzymes in breads made with 50% and 100% tef flour and 100% maize starch bread (control bread). The ANOVA indicated that the tef incorporation level was the only parameter that dictated the level of the starch fractions in the resulting breads. The FSG content in the tef-incorporated breads were relatively higher than that of the control one, which agrees with the earlier findings by Ronda et al. [17] and Shumoy et al. [28] on tef-fortified wheat-based breads. The report by Abebe et al. [12] revealed higher FSG in tef flours than the flours from common cereals like rice and wheat, suggesting this could be the probable reason why cooked tef products have a sweet taste.

The maize starch bread showed higher values of RAG, RDS and TDS fractions than tef-fortified breads, and the values further decreased as the proportion of the tef flours increased from 50% to 100%. This could be due to the intrinsic properties of the type of starch available in tef grain and to the fact that tef flour is a whole flour containing all other grain components (proteins, fiber, lipids), which limits the amount of starch in the flour compared to pure maize starch. This finding agrees with the report on starchy foods that underlines the rapid degradation of starch in the small intestine and very rapid rise of blood glucose level (high glycemic index) because the starches are highly gelatinized and the product structure is very porous [29].

The RDS fraction was the most predominant in all bread samples, which varied from 80 (in 100% maize starch breads) to 58 (in 100% DZ-Cr-37 tef flour breads) g/100 g of bread dry matter, while the SDS fraction varied from 1 to 8 g/100 g, without significant differences among samples, including the control bread. The TDS (TDS = SDS + RDS) contents obtained under this study were closer to the findings of Ronda et al. [17] in 60:40% wheat/tef breads, while they were higher than those reported for crackers made with 100% white tef and 100% brown tef [30]. The formulation of the different types of products and, in particular, their water content could be the source of such differences. Gelatinization of starch, which leads to its rapid digestion, is imminent during the processing of breakfast cereals and bakery products as the starches are heated in the presence of moisture. In contrast, biscuits or crackers are produced by baking under low/very low-moisture conditions, which reduces the extent of starch gelatinization and results in partially intact starch granules that are less susceptible to the action of amylolytic enzymes [31,32]. However, as underlined in Fardet et al. [33], the characteristics not only at the molecular level (degree of starch gelatinization and retrogradation, percentage of amylose) but also at the microscopic level (starch interactions with other food components: proteins, lipids and fibers; food matrix porosity) and at the macroscopic level (food particle size, in particular, its changes during the digestive process) influence the complex process of starch digestibility.

## 4. Conclusions

Tef flour incorporation could be an effective way to enhance the physical, sensorial and nutritional quality of GF maize starch-based breads, depending on both the tef variety and the dose of addition for the formulation. Structuring ability of tef flour was observed in breadmaking performance, affecting all crucial parameters of bread quality. Incorporation of tef flour even at the 50% level significantly improved the mineral content of the resulting GF breads so that the daily requirement of the consumers could be easily satisfied. In addition, breads with a slower starch digestion rate were also obtained. Breads with tef flour had less friable crumb and showed a correct crumb cell structure, without holes or defects. Although the breads made with 100% tef flour resulted in lower specific volumes and greater firmness than those made with lower addition levels, the bread made with 100% DZ-Cr-37 variety achieved the highest sensorial scores for crust and crumb color, odor and texture in the sensory analysis. These results are of great interest to the food industry in its search for high-quality GF products and evidence a successful way of improving the health of GF consumers.

## Figures and Tables

**Table 1:** Effect of tef incorporation level and variety type on the physical quality parameters of GF bread.

Incorporation Level (g/100 g)	Tef Variety	Bake Loss (%)		Specific Volume (mL/g)		Firmness (*N*)		Springiness		Cohesiveness		Chewiness (*N*)		Resilience	
100	DZ-Cr-37	13.3	a	2.03	b	19.6	d	0.85	ab	0.285	b	4.7	c	0.100	ab
	DZ-Cr-387	12.9	a	1.71	a	21.9	e	0.86	ab	0.330	c	6.3	e	0.130	c
	DZ-01-99	16.0	de	2.12	bc	21.3	de	0.93	b	0.273	ab	5.5	d	0.093	ab
75	DZ-Cr-37	15.8	cde	2.50	g	12.7	bc	0.86	ab	0.288	b	3.2	b	0.100	ab
	DZ-Cr-387	17.3	e	2.38	ef	11.7	ab	0.88	ab	0.265	a	2.8	ab	0.093	ab
	DZ-01-99	15.4	bcd	2.29	de	19.5	d	0.87	ab	0.283	ab	5.3	cd	0.100	ab
50	DZ-Cr-37	13.8	ab	2.42	fg	10.9	a	0.84	a	0.270	ab	2.5	a	0.090	a
	DZ-Cr-387	13.7	a	2.20	cd	13.2	c	0.83	a	0.271	ab	2.8	ab	0.090	a
	DZ-01-99	14.2	abc	2.44	fg	10.5	a	0.86	ab	0.270	ab	2.5	a	0.090	a
SE		0.6		0.04		0.7		0.03		0.007		0.2		0.004	
Analysis of variance and significance (*p*-values)															
Tef variety		ns		***		***		ns		*		***		*	
Tef Incorporation level		***		***		***		ns		***		***		***	
Tef variety x Incorporation level		**		***		***		ns		***		***		***	

SE: Pooled standard error. Values with the same letters in a column are not significantly different (*p* > 0.05). Significance level: ***: *p* < 0.001 **: *p* < 0.01 *: *p* < 0.05 ns: not significant.

**Table 2:** Effect of tef incorporation level and variety type on the sensory parameters of GF bread.

Incorporation Level (g/100 g)	Tef Variety	Crust Color		Crumb Color		Odor		Taste		Aftertaste		Texture	
100	DZ-Cr-37	5.5	d	5.6	e	5.8	cd	5.4	ab	4.9	abc	5.5	bc
	DZ-Cr-387	3.6	a	3.3	a	4.3	ab	5.0	ab	4.8	ab	5.5	c
	DZ-01-99	4.3	abc	4.3	abcd	4.5	ab	4.6	ab	4.6	ab	5.1	abc
75	DZ-Cr-37	5.1	bcd	5.3	de	5.3	bcd	5.1	ab	5.3	bc	4.8	abc
	DZ-Cr-387	5.3	cd	5.2	cde	6.1	d	5.6	b	5.3	bc	4.8	abc
	DZ-01-99	4.2	ab	4.1	ab	4.4	ab	4.6	ab	4.6	ab	4.5	abc
50	DZ-Cr-37	4.9	bcd	4.4	bcd	3.7	a	4.3	a	4.2	a	4.3	b
	DZ-Cr-387	4.6	abcd	4.9	bcde	4.3	ab	5.0	ab	5.3	bc	4.7	abc
	DZ-01-99	5.0	bcd	4.6	bcde	5.2	bcd	5.7	b	5.9	c	4.3	a
0	Maize starch	4.4	abcd	4.2	abc	4.8	abc	4.7	ab	4.5	ab	4.4	ab
SE		0.4		0.4		0.5		0.4		0.4		0.4	
Analysis of variance and significance (*p*-values)													
Tef variety		ns		*		ns		ns		ns		ns	
Tef Incorporation level		ns		ns		*		ns		ns		*	
Tef variety x Incorporation level		ns		**		**		ns		*		ns	

SE: Pooled standard error. Values with the same letters in a column are not significantly different (*p* > 0.05). Significance level: ** *p* < 0.01. * *p* < 0.05. ns: not significant.

**Table 3:** Microelement content of tef-enriched GF breads and tef flours and maize starch.

	Incorporation Level (g/100 g)	Tef Variety	Ca mg/100 g		Cr mg/100 g		Cu mg/100 g		Fe mg/100 g		K mg/100 g		Mg mg/100 g		Mn mg/100 g		P mg/100 g		Zn mg/100 g	
Bread	100	DZ-Cr-37	129	c	<0.16		0.32	e	12.5	e	253	c	109	e	2.87	e	246	e	1.6	bcd
		DZ-Cr-387	137	d	<0.16		0.45	g	13.4	f	271	d	105	d	2.24	d	226	d	1.8	cd
		DZ-01-99	144	e	<0.16		0.39	f	9.2	d	281	e	118	f	3.07	f	262	f	2.4	d
	50	DZ-Cr-37	75	b	<0.16		0.18	b	6.6	c	150	b	58	c	1.55	c	142	c	1.1	abc
		DZ-Cr-387	72	b	<0.16		0.24	d	7.1	c	144	b	50	b	1.15	b	120	b	1.1	abc
		DZ-01-99	75	b	<0.16		0.20	c	4.4	b	148	b	56	c	1.57	c	135	c	1.0	ab
	100	Maize starch	10	a	<0.16		<0.16	a	0.3	a	34	a	4	a	<0.16	a	28	a	0.3	a
	SE		2				0.01		0.2		2		1		0.05		2		0.2	
Analysis of variance and significance (*p*-values)																				
Tef variety			ns				ns		ns		ns		ns		ns		ns		ns	
Tef Incorporation level			***				***		***		***		***		***		***		**	
Tef variety x Incorporation level			***				***		***		***		***		***		***		*	
Flour/starch		DZ-Cr-37	255	C	<0.25		0.65	B	25.5	D	478	D	225	D	5.95	C	478	D	2.95	C
		DZ-Cr-387	242	B	<0.25		0.83	D	24.0	C	440	B	185	B	4.21	B	374	B	2.97	C
		DZ-01-99	254	C	<0.25		0.70	C	15.7	B	454	C	208	C	5.84	C	435	C	2.74	B
		Maize starch	1	A	<0.25		<0.25	A	<0.25	A	9	A	2	A	<0.25	A	16	A	<0.25	A
	SE		1				0.01		0.2		3		1		0.06		2		0.05	

SE: Pooled standard error. Within columns, values with the same following letter do not differ significantly from each other (*p* > 0.05). Significance level: *** *p* < 0.001. ** *p* < 0.01. * *p* < 0.05. ns: not significant. Lower case letters are used to compare bread contents and capital letters to compare flour/starch amounts.

**Table 4:** Starch fractions of tef-enriched maize starch breads referred to bread dry matter.

Incorporation Level (g/100 g)	Tef Variety	FSG (g/100 g)		RAG (g/100 g)		RDS (g/100 g)		SDS (g/100 g)		TS (g/100 g)		TDS (g/100 g)	
100	DZ-Cr-37	1.4	b	66	a	58	a	6	a	64	a	64	a
	DZ-Cr-387	1.4	b	71	ab	65	ab	1	a	66	a	66	a
	DZ-01-99	1.1	ab	70	a	62	a	4	a	70	a	66	a
50	DZ-Cr-37	1.1	ab	74	ab	66	abc	8	a	75	b	74	ab
	DZ-Cr-387	1.2	ab	81	b	72	d	5	a	78	b	77	b
	DZ-01-99	1.0	a	79	b	70	cd	5	a	75	b	75	ab
0	Maize starch	0.9	a	90	c	80	e	7	a	86	c	84	b
SE		0.1		3		3		2		2		4	
Analysis of variance and significance (*p*-values)													
Tef variety		ns		ns		ns		ns		ns		ns	
Tef incorporation level		*		**		**		ns		***		**	
Tef variety x Incorporation level		ns		ns		ns		ns		ns		ns	

SE: Standard error. Values with the same letters in a column are not significantly different (*p* > 0.05). Significance level: *** *p* < 0.001. ** *p* < 0.01. * *p* < 0.05. ns: not significant. FSG: Free glucose and glucose from sucrose; RAG = rapidly available glucose; RDS = rapidly digestible starch. SDS = slowly digestible starch; TS = total starch, TDS: total digestible starch.

## Data Availability

All data generated or analyzed during this study are included in this published article.
